# Differential uptake and effects of digital sexually transmitted and bloodborne infection testing interventions among equity-seeking groups: a scoping review

**DOI:** 10.1136/sextrans-2023-055749

**Published:** 2023-07-04

**Authors:** Ihoghosa Iyamu, Rodrigo Sierra-Rosales, Claudia S Estcourt, Amy Salmon, Mieke Koehoorn, Mark Gilbert

**Affiliations:** 1 School of Population and Public Health, The University of British Columbia, Vancouver, British Columbia, Canada; 2 Clinical Prevention Services, British Columbia Centre for Disease Control, Vancouver, British Columbia, Canada; 3 Department of Nursing and Community Health, Glasgow Caledonian University School of Health and Life Sciences, Glasgow, UK; 4 Centre for Health Evaluation and Outcome Sciences, Vancouver, British Columbia, Canada

**Keywords:** health services research, socioeconomic factors, sexual health, reproductive tract infections

## Abstract

**Background:**

Digital sexually transmitted and bloodborne infection (STBBI) testing interventions have gained popularity. However, evidence of their health equity effects remains sparse. We conducted a review of the health equity effects of these interventions on uptake of STBBI testing and explored design and implementation factors contributing to reported effects.

**Methods:**

We followed Arksey and O’Malley’s framework for scoping reviews (2005) integrating adaptations by Levac *et al* (2010). We searched OVID Medline, Embase, CINAHL, Scopus, Web of Science, Google Scholar and health agency websites for peer-reviewed articles and grey literature comparing uptake of digital STBBI testing with in-person models and/or comparing uptake of digital STBBI testing among sociodemographic strata, published in English between 2010 and 2022. We extracted data using the Place of residence, Race, Occupation, Gender/Sex, Religion, Education, Socioeconomic status (SES), Social capital and other disadvantaged characteristics (PROGRESS-Plus) framework, reporting differences in uptake of digital STBBI testing by these characteristics.

**Results:**

We included 27 articles from 7914 titles and abstracts. Among these, 20 of 27 (74.1%) were observational studies, 23 of 27 (85.2%) described web-based interventions and 18 of 27 (66.7%) involved postal-based self-sample collection. Only three articles compared uptake of digital STBBI testing with in-person models stratified by PROGRESS-Plus factors. While most studies demonstrated increased uptake of digital STBBI testing across sociodemographic strata, uptake was higher among women, white people with higher SES, urban residents and heterosexual people. Co-design, representative user recruitment, and emphasis on privacy and security were highlighted as factors contributing to health equity in these interventions.

**Conclusion:**

Evidence of health equity effects of digital STBBI testing remains limited. While digital STBBI testing interventions increase testing across sociodemographic strata, increases are lower among historically disadvantaged populations with higher prevalence of STBBIs. Findings challenge assumptions about the inherent equity of digital STBBI testing interventions, emphasising the need to prioritise health equity in their design and evaluation.

**WHAT IS ALREADY KNOWN ON THIS TOPIC**

- Digital sexually transmitted infection (STI) testing interventions have been promoted as equitable, low-barrier and cost-effective alternatives to clinic-based STI testing.

**WHAT THIS STUDY ADDS**

- This study demonstrates limited evidence of the health equity effects of these interventions. While the interventions increase STI testing across social strata, the increases are lower among historically disadvantaged populations.

**HOW THIS STUDY MIGHT AFFECT RESEARCH, PRACTICE OR POLICY**

- Findings from this study challenge assumptions of the inherent equity of digital STI testing interventions and call attention to health equity-focused design and evaluation of these interventions.

## Background

Over the past decade, digital technologies have been deployed to improve uptake of sexually transmitted and bloodborne infection (STBBI) testing.^1 2^ Digital STBBI testing interventions commonly require people to use websites, mobile apps or other digital media to obtain test requisitions or request postal self-sampling kits without consulting health providers in person.^1^ These interventions are assumed to offer lower-barrier and more cost-effective testing than clinic-based services.^3^ Studies describe multilevel barriers to provider-based testing including long wait times, travel requirements for testing, fear of judgement and other forms of discrimination, and discomfort discussing sexual history with health providers.^3^ These barriers are disproportionately reported among young people, gay and bisexual men who have sex with men (GBMSM) and other populations with higher prevalence of STBBIs.^3^

Despite progress in addressing the global STBBI burden, targets to end epidemics of HIV/AIDS, viral hepatitis B and C, and other STBBIs by 2030 appear to be offtrack, especially among equity-seeking populations.^4^ For instance, 65% of the 1.5 million globally reported annual new cases of HIV occur among key populations (ie, GBMSM, transgender individuals, sex workers, persons who inject drugs and incarcerated people).^4^ In Canada, gonorrhoea and syphilis infections doubled and more than tripled over the last 5 and 10 years, respectively, with greatest increases reported among individuals aged 20–29 years old.^5^ Routine asymptomatic testing for people aged under 30 years and populations with higher prevalence of STBBIs is recommended.^6^ Therefore, public health organisations have implemented digital STBBI testing assuming their convenience, confidentiality and reach inherently promote equitable STBBI testing.^2^

Mechanisms underpinning disparities in the STBBI burden and people’s capacity to leverage digital STBBI interventions have been described.^7^ Among sociodemographic groups, there are differences in access to resources, especially those required to leverage technologies for health influence of their STBBI risk and capacity to use digital interventions, thereby reinforcing or creating new health inequities.^8 9^ These resource disparities are often determined by intersections of minority factors known as PROGRESS-Plus factors (Place of residence, Race/ethnicity, Occupation, Gender, Religion, Education, Socioeconomic status, Social capital, sexual orientation and personal characteristics associated with discrimination including age, features of relationships and time-dependent relationships).^10^ Phelan *et al*’s fundamental causes of health inequalities theory suggests that if interventions effectively reduce inequities, we must expect a ‘give back effect’ where historically disadvantaged populations are more likely to benefit from the intervention (ie, have greater effects) because they initially require services more.^7^ This give back effect characterises equity as opposed to equality which describes equal effects among subpopulations. Exploring differential uptake and effects of digital STBBI testing interventions helps us assess the validity of their health equity assumptions.

Reviews have found only few studies exploring ‘differential effects’ of digital STBBI testing, with under-representation of historically disadvantaged populations among digital STBBI testing service users.^11 12^ However, these reviews were in specific populations like GBMSM,^12^ of specific interventions like online postal self-sampling services^11^ and broader STBBI prevention interventions.^12^ A comprehensive assessment of the health equity impacts of digital STBBI testing is needed. Exploring design and implementation considerations contributing to these impacts may give insights on possible equity-promoting adaptations to current interventions. This review aimed to assess the uptake and differential effects of digital STBBI testing especially among historically disadvantaged populations, exploring design and implementation considerations contributing to observed uptake and effects. Our main research question was, ‘what health equity effects of digital interventions for STBBI testing are reported in the literature?’ We explored this question by asking: (1) what is the relative uptake of digital STBBI testing among minority sociodemographic groups? and (2) what are the differential effects of using digital STBBI interventions? Further, we asked what design and implementation considerations and features contribute to reported health equity effects.

## Methods

### Overview

We conducted a scoping review following Arksey and O’Malley’s framework,^13^ integrating modifications recommended by Levac *et al*.^14^ The review protocol was registered on Open Science Framework.^15^ Our reporting adheres to the Preferred Reporting Items for Systematic Reviews and Meta-analyses (PRISMA) extension for scoping reviews^16^ and the PRISMA-Equity extension.^17^

### Study eligibility criteria

We used the Population/Intervention/Comparators/Outcomes/Time frame framework to define our research questions and study eligibility criteria (table 1). We included primary research investigating the uptake of digital STBBI testing and subsequent positivity, linkage to treatment and/or partner notification rates compared with in-person (provider-based) testing. We included articles describing differential outcomes (as stratified, effect modification or interaction analyses) by any of the PROGRESS-Plus factors. This framework has been used to explore health equity effects of various interventions in systematic reviews.^18^ Given limited number of published articles meeting the criteria, we included articles describing differences in uptake of digital STBBI testing using statistical tests of significance (ie, predictors of uptake using any of the PROGRESS-Plus factors). We included peer-reviewed articles and grey literature published in English between 1 January 2010 and 15 March 2022 (the date of the search). Evidence suggests digital STBBI testing interventions likely to be considered for this review have only been implemented at any scale over the last decade; therefore, our review time frame started from January 2010 to ensure we identified all relevant studies.^11^

**Table 1:** Inclusion and exclusion criteria for selected articles

Domain	Inclusion criteria	Exclusion criteria
Population	People who need STBBI testing. From a health equity perspective, only publications that consider at least one of the PROGRESS-Plus factors as their target population or a stratification level in their analysis or publications.	
Intervention	Any intervention integrating digital technologies to facilitate access to STBBI testing including home testing, self-sampling and/or connections with laboratory services. Web portals including websites with chatbots, etc Applications (mobile and desktop) Social media	Interventions that are mainly in person or clinic based but deliver service notifications via SMS and/or email. Interventions leveraging websites and social media channels that do not provide individualised communication, for example, STBBI information and education portals and promotional websites linked to in-person STBBI testing.
Comparison	No or clinic-based STBBI testing	
Outcome	**Primary outcomes:** uptake of STBBI testing—for example, completion and return of STBBI test samples, frequency of testing and repeat testing rates. **Secondary outcomes:** STBBI positivity rates, linkage to treatment and partner notification rates.	Prevention-related outcomes, for example, condom use, uptake of HIV PrEP, knowledge about STBBIs, etc. Treatment-related outcomes, for example, virological suppression rates in HIV.
Time frame	January 2010 to date	Published before year 2010
Study design	Primary research studies including randomised controlled trials, cohort studies, case–control studies, cross-sectional studies (analytical) and quasi-experimental or pre-intervention and post-intervention study designs or qualitative studies—specifically assessing outcomes of interest. *Included articles must either describe differential outcomes by any of the PROGRESS-Plus factors, stratify effects of digital interventions or conduct effect modification or moderation analysis by and of the PROGRESS-Plus factors.	Reviews and researcher perspectives or commentaries. Editorials, case reports, systematic or scoping reviews, design descriptions of interventions without a primary assessment of outcomes, etc. Papers without full texts available.
Setting	Community settings	Interventions in a contrived or hypothetical setting. Studies of prospective digital interventions that are not implemented or evaluated through the study.
Language	Published in English	Published in any language other than English

PrEP, pre-exposure prophylaxis; PROGRESS-Plus, Place of residence, Race, Occupation, Gender/Sex, Religion, Education, Socioeconomic status, Social capital and other disadvantaged characteristics; SMS, Short Messaging Service; STBBI, sexually transmitted and bloodborne infection.

### Information sources and search

We searched OVID Medline, OVID Embase, CINAHL, Scopus and Web of Science. We also manually searched bibliographies of included studies, and conducted grey literature searches using Google Scholar, and international health agency websites including the WHO, US Centers for Disease Control and European Centre for Disease control websites. In collaboration with a health librarian at the University of British Columbia, we refined our search strategy to capture relevant studies (table 2). We adapted combinations of keywords and medical subheadings terms as appropriate for each database (online supplemental appendix A). We used the PROGRESS-Plus factors known to be related to STBBI (including gender, sex, sexual orientation, age and socioeconomic status) to inform search terms related to health equity. For example, we identified gender minority terms like “transgender”, “women” and “queer” to identify historically disadvantaged populations by gender. We defined digital STBBI testing as any intervention using digital technologies to deliver STBBI testing directly to health service users without needing direct contact with clinics, health providers or any other in-person testing modalities.^2 19^ STBBI search terms were derived from the pan-Canadian framework for STBBIs, including HIV, hepatitis B and C, chlamydia, gonorrhoea, syphilis, human papilloma virus (HPV) and herpes simplex.^6^

10.1136/sextrans-2023-055749.supp1 Supplementary data

**Table 2:** Search terms for scoping review

Sexually transmitted and bloodborne infections*	HIV, HBV, Hepatitis B, HCV, Hepatitis C, chlamydia, gonorrhea, gonorrhoea, syphilis, human papilloma virus, HPV, sexually transmitted infection, herpes genitalis, sexually transmitted, sexually transmitted disease, and blood-borne infection STI, STBBI, STD
Digital interventions†	Online, digital, digital technology, internet-based, web-based, eHealth, mHealth, app, apps, mobile application, smartphone, telemedicine, virtual
Testing	Testing, screening, self-sampling, self-test*, self-collect*, home-testing, diagnos*
Health equity	Health equity, equit*, inequit*, dispari*, equal*, unequal, discriminat*, marginali*, underserved, vulnerab*, disadvantage*, rural, racial*, race, ethnic*, unemploy*, gender, literacy, literate, illitera*, youth, young*, elder*, educational status, educational attainment, educational level, “gay and bisexual men”, gbMSM, homosexual men, sexual minority, stigma*, old*, women, social class, social status, social capital, socioeconomic*, poverty, hard-to-reach.

*Search terms for STBBIs are derived from a combination of related terms identified in the pan-Canadian framework for STBBIs and other similar publications

†*Search terms related to digital interventions are derived from search terms in similar publications*

### Selection procedure

**Figure 1:**
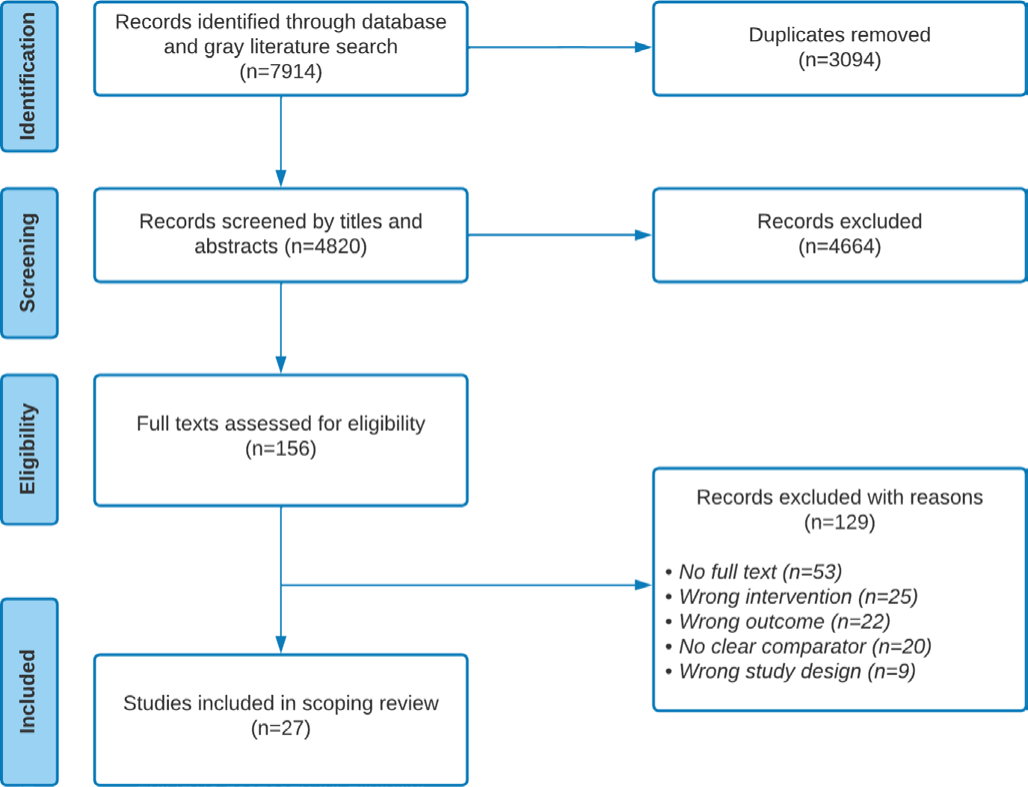
Preferred Reporting Items for Systematic Reviews and Meta-Analyses for Scoping Reviews flow diagram of the search and study selection process.

After importing search returns into Covidence with deduplication, we screened in two phases. First, two reviewers (II and RS-R) independently screened titles and abstracts for relevance. Thereafter, using outlined criteria (table 1), full texts were independently assessed by both reviewers (figure 1). In both phases, whenever there were discrepancies, both reviewers met and discussed until a consensus was achieved. Regarding inter-rater reliability, proportionate agreement was 98% (Cohen’s Κ=0.68) and 92% (Cohen’s Κ=0.75) in the titles/abstract and full-text screening, respectively.

### Empirical critical appraisal of included studies

Considering this was a scoping review, a quality appraisal was not required. However, we assessed the quality of the available evidence using the Mixed Methods Appraisal Tool^20^ (online supplemental appendix B).

### Data charting process

Data from included articles were extracted using pretested data extraction forms on Covidence (online supplemental appendix C). In addition to bibliographical data including authors’ names, year of publication and country of the first author, we extracted our primary outcomes of interest (ie, uptake of STBBI testing including rates of testing completion or return of STBBI test samples, frequency of testing and repeat testing) and secondary outcomes (ie, STBBI test positivity rates, treatment linkage and partner notification rates). Outcomes were extracted using the PROGRESS-Plus framework.^10 17 21^ We reported the types of digital technologies implemented in the intervention (eg, web-based portals, social media, live chat with human staff or chatbots, mobile applications, video-assisted STBBI testing services, vending machines, virtual reality and/or a combination of the above). We also explored design and implementation factors suggested by study authors as explanations for observed differential outcomes.

### Synthesis of results

We summarised the data using simple descriptive statistics and a narrative synthesis based on the PROGRESS-Plus factors.^21^ We also summarised design and implementation factors contributing to observed outcomes.

## Results

### Characteristics of included papers and digital interventions

Table 3 describes the 27 studies included in this review. Of these, 7 (25.9%) were from the USA, 13 (48.2%) were published between 2015 and 2019, 20 (74.1%) were observational studies, 6 (22.2%) were randomised controlled trials and 1 study highlighted theoretical underpinnings of the intervention. Regarding digital technologies implemented, 23 (85.2%) described interventions involving web-based portals (ie, sample requisition or request for self-sampling kits via a website), 2 (7.4%) described interventions combining video-assisted testing and digital personal health records, while 1 (3.7%) was implemented as a mobile app. Overall, 18 studies (66.7%) described interventions using online postal self-sample collection, 6 (22.2%) described self-sample collection and interpretation of results (ie, self-testing) and 2 (7.4%) described interventions using online test requisition with specimen submission at a laboratory. The quality of included studies was variable, with majority using observational (post-hoc) study designs (online supplemental appendix B).

**Table 3:** Characteristics of included articles

Characteristic	Frequency (n=27)	Per cent (100%)
Country of first author		
USA	7	25.9
UK	6	22.2
The Netherlands	5	18.5
China	3	11.1
France	3	11.1
Thailand	2	7.4
Canada	1	3.7
Year of publication		
2010–2014	8	29.6
2015–2019	13	48.2
2020–2021*	6	22.2
Type of study		
Study design		
Observational study	20	74.1
Non-randomised experimental study	1	3.7
Randomised controlled trial	6	22.2
Type of digital intervention		
Web-based testing	23	85.2
Video-assisted testing services, web-based testing and electronic health records	2	7.4
Mobile applications	1	3.7
Social media	1	3.7
Sample collection methods		
Postal-based self-sample collection	18	66.7
Self-sample collection and interpretation	6	22.2
Lab-assisted sample collection	2	7.4
Self-sample collection and interpretation; self-sample collection and postal	1	3.7

*Only includes 2 years of data compared with 5 years in other categories.

### Consideration of PROGRESS-Plus factors in analyses of the impact of digital interventions

The studies operationalised the PROGRESS-Plus factors in varying ways (online supplemental appendix D). Most (24 of 27) explored users’ age, 19 of 27 explored gender either as baseline characteristics or as factors influencing uptake of digital STBBI testing, while 13 of 27 explored place of residence, measured either as city names or sizes. Only 6 of 27 studies explored concepts related to occupation (mainly as employment status), while 14 of 27 studies described participants’ education. Conceptions of gender identity and sexual orientation were explored in 19 of 27 studies, but categories were not standardised (eg, including all transgender people under the same category as sexual orientation, or separating ‘gay’ from ‘males’ under a gender identity category).^22–27^ Socioeconomic status was measured in 13 of 27 studies using different constructs, including income, self-assessed financial situation or Index of Multiple Deprivation (IMD). No study assessed religion or social capital. Some studies considered additional sociodemographic factors including possession of health insurance, marital status, housing status and place of birth.^28 29^

### Overall impacts of digital interventions for STBBI testing compared with clinic-based testing

Most studies comparing digital STBBI testing with clinic-based testing described higher uptake of STBBI testing through digital STBBI testing interventions,^22 24 30–32^ reduced time to testing^32 33^ and higher positivity rates (ie, number of people testing positive for any STBBI per tests conducted) (online supplemental appendix E summarises findings from included studies).^22^ Some studies described insignificant differences in uptake^34^ and positivity rates between digital and clinic-based STBBI testing,^30 32 35^ while one study demonstrated higher uptake of clinic-based testing and positivity rates compared with digital STBBI testing.^26^

### Differences in sociodemographic (PROGRESS-Plus) characteristics of people using digital interventions for STBBI testing

Most studies found women,^30 31 36–42^ users aged over 20 years,^25 36 38 39 41 43 44^ higher-income earners,^37 39 41 42^ white non-Hispanic people,^25 29 31 35 41 45 46^ people with origins within the jurisdiction where the intervention was implemented,^30 37 39 40 42^ heterosexual people,^31^ cis-gender individuals,^25^ residents of urbanised areas,^36 37^ and those with college level or higher education were more likely to use digital STBBI testing.^25 40 43 45 46^ People with health insurance were also reported as more likely to use digital STBBI testing in one study.^29^ Conversely, some studies found lower-income earners,^22^ people in less urbanised regions,^43 43^ males,^35^ transgender and non-binary people,^47^ and GBMSM had higher likelihood of uptake of digital STBBI testing.^29 41^ Others did not find any sociodemographic factors associated with digital STBBI testing.^23 27^

### Differential effects of digital interventions for STBBI testing stratified by PROGRESS-Plus factors

Only three studies conducted a direct comparison of outcomes for digital and in-person STBBI testing stratified by PROGRESS-Plus factors or investigated differences by these factors using subgroup or interaction analyses, and all three were randomised controlled trials.^30 32 33^ One study examined the effect of digital interventions for STBBI stratified by gender,^30^ while the two examined effects across gender, sexual orientation, ethnicity, age and IMD (socioeconomic status).^32 33^ Overall, all three studies demonstrated higher uptake of STBBI testing through digital STBBI testing compared with in-person testing across all sociodemographic strata. One study demonstrated significantly higher uptake of digital STBBI testing among men compared with women,^30^ while two studies showed that women had higher (but insignificant) rates of uptake of digital STBBI testing than males.^32 33^ Two studies assessed adjusted testing completion rates (ie, rates of completing a test through the service among the general population of people invited to the study) and found that women had significantly higher completion rates than men.^36 37^ Regarding age, three studies reported increasing rates of uptake of digital STBBI testing with increasing age, although differences between age groups were not statistically significant.^32 33 37^ One study reported similarly higher uptake of digital STBBI testing among white and black/African/Caribbean users,^32^ while another study among people who had never tested for STBBI showed statistically significant higher uptake of digital STBBI testing for white but non-significant effects for black/African/Caribbean and Asian users.^33^ However, the sample size was limited for subgroup analyses in this case.^33^ Regarding sexual orientation, two studies demonstrated lower rates of digital STBBI testing for MSM compared with other people and increasing rates of digital STBBI testing with lower levels of deprivation (ie, higher socioeconomic status).^32 33^

### Design and implementation factors contributing to health equity effects of digital interventions for STBBI testing

Eight studies commented on design and implementation factors contributing to observed differential impacts of their digital STBBI testing interventions that foster health equity. Three studies highlighted the role of personalised reminders in engaging users,^31 36 40^ while one described strategic needs-based expansion of services into new geographical areas known to have more deprived populations.^36^ Two studies were based on interventions that were co-designed and implemented by community organisations led by people who experience marginalisation to build trust among those requiring services.^27 40^ Two studies described the importance of ensuring appropriate representation in the recruitment materials (ie, in a study context), especially for people of colour, and emphasised the role of local health authorities in media campaigns.^25 43^ Others described the importance of emphasising privacy features and the availability of the service outside traditional clinic hours in promotional material.^26 28^ One study described using prior education of potential users to ensure their understanding of the service.^40^

Four studies described features of their digital STBBI testing interventions explaining why people facing sexual health disparities may not benefit to the same extent as others. Two studies suggested that the unavailability of additional health services alongside digital STBBI testing, including contraception, hepatitis B virus and HPV vaccination services, may explain users’ preference for clinic-based STBBI testing.^34 41^ One study suggested that online postal self-sampling services were unable to reach people with non-standardised mailbox services,^48^ while another suggested that reliance on online channels for promotion and user recruitment may restrict use to people with higher levels of education.^46^

## Discussion

Our study contributes evidence about the differential effects of digital STBBI testing interventions among various equity-seeking groups. It challenges health equity assumptions underpinning these interventions, highlighting the importance of equity-focused implementation and evaluation. While studies reported on sociodemographic groups using digital STBBI testing, only few studies explored differential effects of these interventions. We found higher rates of digital STBBI testing compared with in-person testing among white people, women, people with higher socioeconomic status, urban residents and people with other sexual orientations compared with GBMSM. Similar patterns were observed on likelihood of using digital STBBI testing interventions as users were more likely to be white, non-Hispanic women (females), older adults and urban residents. Digital STBBI testing interventions were found to increase uptake of STBBI testing across most PROGRESS-Plus factors explored. However, the uptake of digital STBBI testing was disproportionately lower for historically disadvantaged groups with higher prevalence of STBBIs. Few studies commented on design and implementation factors contributing to health equity effects. Co-design, representative recruitment, emphasis on privacy and security measures of the digital interventions were suggested as important factors.

Our findings are congruent with a review of online postal sexual services in the UK which suggests under-representation of populations bearing disproportionate burdens of STBBIs among users of digital STBBI testing interventions.^11^ Other systematic reviews have similarly demonstrated benefits of digital interventions promoting STBBI testing.^12 49^ Among GBMSM and transgender women, one systematic review reported increased STBBI testing with digital interventions including health promotion applications.^49^ However, our findings extend existing literature by describing subgroup differences in uptake despite increased uptake overall. Our review suggests that uptake among disadvantaged populations most in need of STBBI testing lags other populations. Another review on digital patient health portals further suggests inadequate attention to digital health equity, and a disproportionate focus on individual-level barriers and facilitators contributes to disparities.^50^ Less attention to systems-level issues influencing uptake and considerations of the impact of users’ social positions on uptake has reinforced an individualistic view of the problems.^50^ Similarly, our review found no reference to users’ social positions or social capital when considering health equity factors influencing uptake of these services. Difficulties operationalising social capital in routine data collection may be a plausible reason for this observation.

Practitioners have only recently begun pivoting from implementing digital health interventions based on technological optimism (non-critical belief that technology inevitably solves all problems) and determinism (beliefs that technology is the principal initiator of society’s transformation).^8^ Prior optimism may explain the paucity of evidence on health equity effects of digital STBBI testing interventions. Differences in health-seeking behaviours may explain some differential effects identified in our study. However, differences in access to flexible resources more appropriately explain persistent disparities in uptake of digital STBBI testing interventions.^7^ Researchers assert that interventions can be designed and implemented to minimise the role flexible resources play.^7^ Digital health interventions have not yet achieved this goal, as they require users to have significant access to material circumstances and digital health literacy required to effectively leverage such interventions.^8^

These findings emphasise the need for equity-focused design, implementation and evaluation of digital STBBI testing interventions.^8^ They also highlight concerns about the risk for digital interventions to reinforce and create new inequities.^8 9^ Our findings challenge widely held beliefs that digital STBBI testing interventions are the most effective way to reduce inequities, especially for historically disadvantaged populations, bearing disproportionate burdens of STBBIs.^2^ While being effective in reaching many population subgroups,^32^ our review suggests that more systematic approaches to design and implementation of these interventions may be needed if those most affected by STBBIs are to equitably benefit.^8 9^ Strategies like ensuring representation in promoting interventions, co-designing interventions with most affected populations, adapting to user needs and communicating privacy measures undertaken within interventions to allay community concerns have been described in the literature.^8 9^ Harnessing behaviour change theory may also be useful in systematically optimising the design and implementation of STBBI testing interventions. We must emphasise that these strategies must concurrently occur alongside larger societal efforts to guarantee equitable access to resources required to effectively use digital interventions.^7 8^

Our findings of incomplete reporting of effects across PROGRESS-Plus factors further highlight inadequate attention to health equity in evaluating digital STBBI testing interventions. Equity-focused researchers recommend the application of health equity lens in these evaluations using pre-hoc specified, stratified, subgroup or interaction analyses.^10^ Implementation of such recommendations, especially using equity frameworks like the PROGRESS-Plus framework, can help practitioners understand intended and unintended consequences of their interventions among various subpopulations. Such equity-focused analyses may also inform more thoughtful interventions grounded in users’ realities. Further research on design and implementation factors responsible in observed differential effects can inform better understanding of issues encountered with these interventions. Further research is also required to determine if uptake of these interventions by all sociodemographic groups frees up capacity for in-clinic (provider-based) testing, reducing barriers to these services.

### Strengths and limitations of the study

To the best of our knowledge, this is the first review of evidence on digital STBBI testing that explicitly explores health equity as compared with others that have considered their differential effects in a wider context.^12^ Our use of the PROGRESS-Plus framework offers a systematic approach to searching the literature, extracting data, identifying and analysing relevant equity impacts of equity-focused interventions. However, the review is limited by our broad definition of health equity effects, as we assessed studies that conducted subgroup, interaction or stratified analyses, and studies exploring how PROGRESS-Plus factors influenced uptake of digital STBBI testing. Our broad approach to health equity may have introduced heterogeneity into our review, but it was necessary given we anticipated few equity-focused studies within the literature. Our search was limited to between January 2010 and March 2022. While evidence supports this period as relevant for digital STBBI interventions, our stringent criteria may have inadvertently missed some studies. We also considered only publications in English, which may have unintentionally excluded relevant studies. The limited number of studies also reduced our ability to assess differences in impacts of digital STBBI testing interventions that used different digital technologies and sample collection methods.

## Conclusion

Evidence about the health equity impacts of digital STBBI testing interventions remains limited. Knowledge gaps remain regarding differences in uptake of digital STBBI testing among PROGRESS-Plus subgroups. The limited evidence suggests digital STBBI testing interventions increase STBBI testing across various sociodemographic strata. However, these increases are less for populations disproportionately bearing the burden of STBBIs, including GBMSM, people of colour and youth. This evidence challenges widely held beliefs that digital STBBI testing interventions ultimately improve health equity by eliminating barriers to clinic-based testing. Design and implementation considerations highlighted as important in reaching historically disadvantaged groups include co-design, representative recruitment, emphasis on privacy and security measures of the digital interventions. Implementers and researchers must resist technologically optimistic and deterministic thinking when addressing inequities in access to health services. Rather, we must prioritise equity-focused design, implementation and systematic evaluation of digital STBBI testing interventions to improve their public health benefit.

## Data Availability

All data relevant to the study are included in the article or uploaded as supplemental information.
