# Supplementary material for: Differential uptake and effects of digital sexually transmitted and bloodborne infection testing interventions among equity-seeking groups: a scoping review
Source: Sex Transm Infect. 2023 Jul 4;99(8):554–60. doi: 10.1136/sextrans-2023-055749 (PMC10715539; doi:10.1136/sextrans-2023-055749)
Supplement: Supplementary data [file sextrans-2023-055749supp001.pdf]

Appendices

Appendix A – Search strategy per database

Ovid Embase search

Database:
Embase <1974 to 2022 March 14>

#	Query	Results from 15 Mar 2022
1	blood-borne infections/ or exp hepatitis b/ or exp hepatitis c/ or exp hiv infections/ or exp sexually transmitted diseases/	672,946
2	(HIV or HBV or Hepatitis B or HCV or Hepatitis C or chlamydia or gonorrhea or gonorrhoea or syphilis or human papilloma virus or HPV or sexually transmitted infection or sexually transmitted diseases or herpes genitalis or sexually transmitted disease or blood-borne infection or blood-borne infections or STI or STBBI or STD).mp. [mp=title, abstract, heading word, drug trade name, original title, device manufacturer, drug manufacturer, device trade name, keyword heading word, floating subheading word, candidate term word]	879,041
3	1 or 2	1,016,615
4	exp Telemedicine/ or exp Internet/	173,670
5	(Online or digital or digital technology or internet-based or web-based or eHealth or mHealth or app or apps or mobile application or smartphone or telemedicine or virtual).mp. [mp=title, abstract, heading word, drug trade name, original title, device manufacturer, drug manufacturer, device trade name, keyword heading word, floating subheading word, candidate term word]	716,893
6	4 or 5	799,450
7	exp HIV Testing/ or exp Point-of-Care Testing/ or exp Self-Testing/	31,301
8	(Testing or screening or self-sampling or self-test* or self-collect* or home-testing or diagnos*).mp. [mp=title, abstract, heading word, drug trade name, original title, device manufacturer, drug manufacturer, device trade name, keyword heading word, floating subheading word, candidate term word]	8,353,065
9	7 or 8	8,354,855
10	(Health equity or equit* or inequit* or dispari* or equal* or unequal or discriminat* or marginali* or underserved or vulnerab* or disadvantage* or rural or racial* or race or ethnic* or unemploy* or gender or literacy or literate or illitera* or youth or young* or elder* or educational status or educational attainment or educational level or gay men or bisexual men or gbMSM or homosexual men or sexual minority or transgender or queer or lesbian or bisexual or two-spirit or intersex or stigma* or old* or women	6,926,903

	or social class or social status or social capital or socioeconomic* or poverty or hard-to-reach).mp. [mp=title, abstract, heading word, drug trade name, original title, device manufacturer, drug manufacturer, device trade name, keyword heading word, floating subheading word, candidate term word]	
11	exp Health Equity/ or exp Health Status Disparities/ or exp Socioeconomic Factors/	469,146
12	10 or 11	7,020,460
13	3 and 6 and 9 and 12	3,947
14	exp "Review Literature as Topic"/ or exp "Systematic Review"/ or exp "Ethical Review"/ or scoping review.mp.	885,211
15	13 not 14	3,720
16	protocol.mp. or Clinical Trial Protocol/	638,047
17	15 not 16	3,588
18	limit 17 to (english language and yr="2010 -Current")	3,270

blood-borne infections/ or exp hepatitis b/ or exp hepatitis c/ or exp hiv infections/ or exp sexually transmitted diseases/
(HIV or HBV or Hepatitis B or HCV or Hepatitis C or chlamydia or gonorrhea or gonorrhoea or syphilis or human papilloma virus or HPV or sexually transmitted infection or sexually transmitted diseases or herpes genitalis or sexually transmitted disease or blood-borne infection or blood-borne infections or STI or STBBI or STD).mp. [mp=title, abstract, heading word, drug trade name, original title, device manufacturer, drug manufacturer, device trade name, keyword heading word, floating subheading word, candidate term word]
1 or 2
exp Telemedicine/ or exp Internet/
(Online or digital or digital technology or internet-based or web-based or eHealth or mHealth or app or apps or mobile application or smartphone or telemedicine or virtual).mp. [mp=title, abstract, heading word, drug trade name, original title, device manufacturer, drug manufacturer, device trade name, keyword heading word, floating subheading word, candidate term word]
4 or 5
exp HIV Testing/ or exp Point-of-Care Testing/ or exp Self-Testing/
(Testing or screening or self-sampling or self-test* or self-collect* or home-testing or diagnos*).mp. [mp=title, abstract, heading word, drug trade name, original title, device manufacturer, drug manufacturer, device trade name, keyword heading word, floating subheading word, candidate term word]
7 or 8
(Health equity or equit* or inequit* or dispari* or equal* or unequal or discriminat* or marginali* or underserved or vulnerab* or disadvantage* or rural or racial* or race or ethnic* or unemploy* or gender or literacy or literate or illitera* or youth or young* or elder* or educational status or educational attainment or educational level or gay men or bisexual men or gbMSM or homosexual men or sexual minority or transgender or queer or lesbian or bisexual or two-spirit or intersex or stigma* or old* or women or social class or social status or social capital or socioeconomic* or poverty or hard-to-

reach).mp. [mp=title, abstract, heading word, drug trade name, original title, device manufacturer, drug manufacturer, device trade name, keyword heading word, floating subheading word, candidate term word]
exp Health Equity/ or exp Health Status Disparities/ or exp Socioeconomic Factors/
10 or 11
3 and 6 and 9 and 12
exp "Review Literature as Topic"/ or exp "Systematic Review"/ or exp "Ethical Review"/ or scoping review.mp.
13 not 14
protocol.mp. or Clinical Trial Protocol/
15 not 16
limit 17 to (english language and yr="2010 -Current")

https://access.ovid.com/custom/redirector/index.html?dest=https://go.openathens.net/redirector/ubc.ca?url=http://ovidsp.ovid.com/ovidweb.cgi?T=JS&NEWS=N&PAGE=main&SHAREDSEARCHID=4gzXDxqkn1ayMnb0QgTnQQyADlUy3vaKPsFOucHfj15bHhUrbjKTesaFEufsfMV47

Ovid Medline search
Database:
Ovid MEDLINE(R) and Epub Ahead of Print, In-Process, In-Data-Review & Other Non-Indexed Citations, Daily and Versions <1946 to March 14, 2022>

#	Query	Results from 15 Mar 2022
1	blood-borne infections/ or exp hepatitis b/ or exp hepatitis c/ or exp hiv infections/ or exp sexually transmitted diseases/	481,028
2	(HIV or HBV or Hepatitis B or HCV or Hepatitis C or chlamydia or gonorrhea or gonorrhoea or syphilis or human papilloma virus or HPV or sexually transmitted infection or sexually transmitted diseases or herpes genitalis or sexually transmitted disease or blood-borne infection or blood-borne infections or STI or STBBI or STD).mp. [mp=title, abstract, original title, name of substance word, subject heading word, floating sub-heading word, keyword heading word, organism supplementary concept word, protocol supplementary concept word, rare disease supplementary concept word, unique identifier, synonyms]	688,294
3	1 or 2	732,480
4	exp Telemedicine/ or exp Internet/	125,924
5	(Online or digital or digital technology or internet-based or web-based or eHealth or mHealth or app or apps or mobile application or smartphone or telemedicine or virtual).mp. [mp=title, abstract, original title, name of substance word, subject heading word, floating sub-heading word, keyword heading word, organism supplementary concept word, protocol supplementary concept word, rare disease supplementary concept word,	510,614

Iyamu I, *et al. Sex Transm Infect* 2023; 99:554–560. doi: 10.1136/sextrans-2023-055749

	unique identifier, synonyms]	
6	4 or 5	562,990
7	exp HIV Testing/ or exp Point-of-Care Testing/ or exp Self-Testing/	19,245
8	(Testing or screening or self-sampling or self-test* or self-collect* or home-testing or diagnos*).mp. [mp=title, abstract, original title, name of substance word, subject heading word, floating sub-heading word, keyword heading word, organism supplementary concept word, protocol supplementary concept word, rare disease supplementary concept word, unique identifier, synonyms]	6,424,054
9	7 or 8	6,431,099
10	(Health equity or equit* or inequit* or dispari* or equal* or unequal or discriminat* or marginali* or underserved or vulnerab* or disadvantage* or rural or racial* or race or ethnic* or unemploy* or gender or literacy or literate or illitera* or youth or young* or elder* or educational status or educational attainment or educational level or gay men or bisexual men or gbMSM or homosexual men or sexual minority or transgender or queer or lesbian or bisexual or two-spirit or intersex or stigma* or old* or women or social class or social status or social capital or socioeconomic* or poverty or hard-to-reach).mp. [mp=title, abstract, original title, name of substance word, subject heading word, floating sub-heading word, keyword heading word, organism supplementary concept word, protocol supplementary concept word, rare disease supplementary concept word, unique identifier, synonyms]	5,433,010
11	exp Health Equity/ or exp Health Status Disparities/ or exp Socioeconomic Factors/	498,405
12	10 or 11	5,567,546
13	3 and 6 and 9 and 12	2,321
14	exp "Review Literature as Topic"/ or exp "Systematic Review"/ or exp "Ethical Review"/ or scoping review.mp.	219,606
15	13 not 14	2,260
16	protocol.mp. or Clinical Trial Protocol/	392,509
17	15 not 16	2,126
18	limit 17 to (english language and yr="2010 -Current")	1,900

blood-borne infections/ or exp hepatitis b/ or exp hepatitis c/ or exp hiv infections/ or exp sexually transmitted diseases/
(HIV or HBV or Hepatitis B or HCV or Hepatitis C or chlamydia or gonorrhea or gonorrhoea or syphilis or human papilloma virus or HPV or sexually transmitted infection or sexually transmitted diseases or herpes genitalis or sexually transmitted disease or blood-borne infection or blood-borne infections or STI or STBBI or STD).mp. [mp=title, abstract, original title, name of substance word, subject heading word, floating sub-heading word, keyword heading word, organism supplementary concept word, protocol supplementary concept word, rare disease supplementary concept word, unique identifier, synonyms]

1 or 2
exp Telemedicine/ or exp Internet/
(Online or digital or digital technology or internet-based or web-based or eHealth or mHealth or app or apps or mobile application or smartphone or telemedicine or virtual).mp. [mp=title, abstract, original title, name of substance word, subject heading word, floating sub-heading word, keyword heading word, organism supplementary concept word, protocol supplementary concept word, rare disease supplementary concept word, unique identifier, synonyms]
4 or 5
exp HIV Testing/ or exp Point-of-Care Testing/ or exp Self-Testing/
(Testing or screening or self-sampling or self-test* or self-collect* or home-testing or diagnos*).mp. [mp=title, abstract, original title, name of substance word, subject heading word, floating sub-heading word, keyword heading word, organism supplementary concept word, protocol supplementary concept word, rare disease supplementary concept word, unique identifier, synonyms]
7 or 8
(Health equity or equit* or inequit* or dispari* or equal* or unequal or discriminat* or marginali* or underserved or vulnerab* or disadvantage* or rural or racial* or race or ethnic* or unemploy* or gender or literacy or literate or illitera* or youth or young* or elder* or educational status or educational attainment or educational level or gay men or bisexual men or gbMSM or homosexual men or sexual minority or transgender or queer or lesbian or bisexual or two-spirit or intersex or stigma* or old* or women or social class or social status or social capital or socioeconomic* or poverty or hard-to-reach).mp. [mp=title, abstract, original title, name of substance word, subject heading word, floating sub-heading word, keyword heading word, organism supplementary concept word, protocol supplementary concept word, rare disease supplementary concept word, unique identifier, synonyms]
exp Health Equity/ or exp Health Status Disparities/ or exp Socioeconomic Factors/
10 or 11
3 and 6 and 9 and 12
exp "Review Literature as Topic"/ or exp "Systematic Review"/ or exp "Ethical Review"/ or scoping review.mp.
13 not 14
protocol.mp. or Clinical Trial Protocol/
15 not 16
limit 17 to (english language and yr="2010 -Current")

https://access.ovid.com/custom/redirector/index.html?dest=https://go.openathens.net/redirector/ubc.ca?url=http://ovidsp.ovid.com/ovidweb.cgi?T=JS&NEWS=N&PAGE=main&SHAREDSEARCHID=4Au3KW8BcR6TX99TOI9FHRpHraC1GYPMBfPaP8HeisFjkSzZkwDCVhRyqWOh21O54

Scopus search

(KEY (health AND equity OR equit* OR inequit* OR dispari* OR equal* OR unequal OR disc riminat* OR marginali* OR underserved OR vulnerab* OR disadvantage* OR rural OR racial* O R race OR ethnic* OR unemploy* OR gender OR literacy OR literate OR illitera* OR youth O R young* OR elder* OR educational AND status OR educational AND attainment OR education al AND level OR gay AND men OR bisexual AND men OR gbmsm OR homosexual AND me

n OR sexual AND minority OR transgender OR queer OR lesbian OR bisexual OR two-spirit OR intersex OR stigma* OR old* OR women OR social AND class OR social AND statu s OR social AND capital OR socioeconomic* OR poverty OR hard-to-reach) AND KEY (testing OR screening OR self-sampling OR self-test* OR self-collect* OR home-testing OR diagnos*) AND KEY (online OR digital OR digital AND technology OR internet-based OR web-based OR ehealth OR mhealth OR app OR apps OR mobile AND application OR smartphone O R telemedicine OR virtual) AND KEY (hiv OR hbv OR hepatitis AND b OR hcv OR hepatitis AND c OR chlamydia OR gonorrhea OR gonorrhoea OR syphilis OR human AND papilloma A ND virus OR hpv OR sexually AND transmitted AND infection OR sexually AND transmitted AND diseases OR herpes AND genitalis OR sexually AND transmitted AND disease OR blood-borne AND infection OR blood-borne AND infections OR sti OR stbbi OR std)) AND PUBYEAR > 2009

Web of science search

(ALL=(HIV or HBV or Hepatitis B or HCV or Hepatitis C or chlamydia or gonorrhea or gonorrhoea or syphilis or human papilloma virus or HPV or sexually transmitted infection or sexually transmitted diseases or herpes genitalis or sexually transmitted disease or blood-borne infection or blood-borne infections or STI or stbbi or STD) AND ALL=(Online or digital or digital technology or internet-based or web-based or eHealth or mHealth or app or apps or mobile application or smartphone or telemedicine or virtual) AND ALL=(Testing or screening or self-sampling or self-test* or self-collect* or home-testing or diagnos*) AND ALL=(Health equity or equit* or inequit* or dispari* or equal* or unequal or discriminat* or marginali* or underserved or vulnerab* or disadvantage* or rural or racial* or race or ethnic* or unemploy* or gender or literacy or literate or illitera* or youth or young* or elder* or educational status or educational attainment or educational level or gay men or bisexual men or gbmsm)) NOT ALL=(systematic review or protocol or scoping review)

Google scholar strategy: March 15
digital OR online OR internet-based OR web-based OR eHealth OR mHealth OR mobile OR smartphone OR telemedicine test OR OR OR testing OR OR OR screening OR OR OR equity OR OR OR Health OR equity "sexually transmitted infection"

Appendix B: MMAT Quality appraisal of included articles

Author (Date)	S1. Are there clear research questions?	S2. Do the collected data allow to address the research questions?	2.1 Is randomization appropriately performed?	2.2 Are the groups comparable at baseline?	2.3 Are there complete outcome data?	2.4 Are the outcome assessors blinded to the intervention provided?	2.5 Did the participants adhere to the assigned intervention?	Comments
VanDenBroek 2012	Yes	Yes	Can't tell	No	Yes	Yes	Yes	
Kersaudy-Rahib 2017	Yes	Yes	Yes	Yes	Yes	Can't tell	Yes	
Wilson 2017	Yes	Yes	Yes	Yes	Yes	Yes	Yes	Missing data imputation helped create complete outcome data
Wilson 2019	Yes	Yes	Yes	Yes	Yes	Can't tell	Yes	Sub-analysis of original RCT also included in this review.
MacGowan 2020	Yes	Yes	Can't tell	Yes	No	Can't tell	Yes	
**Author (Date)**	**S1. Are there clear research questions?**	**S2. Do the collected data allow to address the research questions?**	**3.1 Are the participants representative of the target population?**	**3.2 Are the measurements appropriate regarding both the outcome and intervention (or exposure)?**	**3.3 Are there complete outcome data?**	**3.4 Are the confounders accounted for in the design and analysis?**	**3.5 During the study period, is the intervention administered (or exposure occurred) as intended?**	**Comments**
Fistonich 2021	Yes	Yes	Can't tell	Yes	No	No	Yes	Secondary outcome had response of less

								than 33%
Van Bergen 2010	Yes	Yes	Yes	Yes	Yes	Can't tell	Yes	Stratification is done by age and gender, but other confounders excluded from analyses
Zuure 2011	Yes	Yes	Can't tell	Yes	No	Yes	Yes	Limited information about those not completing the process before receiving lab test orders. These individuals may differ enough to alter estimates differential uptake
Jenkins 2011	Yes	Yes	No	Yes	Yes	No	Yes	Although no inclusion and exclusion criteria were clearly stated, the biases in the promotion as noted mean that the sample is not exactly representative.
Op de Coul 2012	Yes	Yes	Yes	Yes	Yes	Yes	Yes	

Iyamu I, *et al. Sex Transm Infect* 2023; 99:554–560. doi: 10.1136/sextrans-2023-055749

Gotz 2013	Yes	Yes	Yes	Yes	Yes	Yes	Yes	Given that all population was exposed to the intervention with no control group, any other event or co-intervention that could have occurred happened for all population as well. There was no change in the intervention in time as described by the authors.
Gaydos 2013	Yes	Yes	Yes	Yes	Yes	Yes	Yes	
Spielberg 2014	Yes	Yes	Yes	Yes	Yes	Yes	Yes	
Ricca 2016	Yes	Yes	Yes	Yes	Yes	Yes	Can't tell	They mentioned that the samples may not be that of the participants
Anand 2017	Yes	Yes	Can't tell	Yes	Yes	No	Yes	
Lydie 2017	Yes	Yes	No	Yes	No	Yes	Yes	
Manavi 2017	Yes	Yes	Yes	Yes	Yes	Yes	Yes	
Barnard 2018	Yes	Yes	Yes	Yes	Yes	Yes	Yes	
Banerjee 2018	Can't tell	Yes	Yes	Yes	Yes	No	Yes	

Iyamu I, *et al. Sex Transm Infect* 2023; 99:554–560. doi: 10.1136/sextrans-2023-055749

Phanuphak 2018	Yes	Yes	Can't tell	Yes	Yes	Yes	Yes	We do not know what they promoted on social media. People that might have been interested in the fully online option may not have enrolled if the study promoted research on HIV testing preferences without specifying the options available.
Grov 2019	Yes	Yes	Can't tell	Yes	Yes	Yes	Yes	
Gilbert 2019	Yes	Yes	Yes	Yes	Yes	Yes	Yes	
Yan 2020	Yes	Yes	Yes	Yes	Yes	Yes	Yes	
Wu 2021	Yes	Yes	No	Yes	Yes	No	Yes	
Rahib 2021	Yes	Yes	No	Yes	Yes	Yes	Yes	
Chan 2021	Yes	Yes	Yes	Yes	Yes	Yes	Yes	
Day 2021	Yes	Yes	Can't tell	Yes	Yes	No	Yes	

Notes:

Iyamu I, *et al. Sex Transm Infect* 2023; 99:554–560. doi: 10.1136/sextrans-2023-055749

Appendix C - Data Extraction Form for scoping review (blank)

Systematic Review Data Extraction Form

ID no. (as assigned in Covidence)	
Citation:	
Article type:	
Country (what is the country where the institution of the first author is located?):	
Year of publication:	
Completed by:	

Summary Notes

Study Description

Study aims/research questions	
Rationale (what are the stated reasons for the study in the particular population?)	
Participants (number and description – describe the strata where applicable by the PROGRESS-Plus factors) - indicate NA where data is unavailable	
• Place of residence	
• Race/Ethnicity	
• Occupation	
• Gender	
• Religion	
• Educational attainment/Level	
• Sexual orientation	
• Socio-economic status	
• Social capital	
• Age	
• Others (related to PROGRESS-Plus)	
Setting (geographic location, institutional setting, other place/space-related features)	
Name of the digital health intervention	
Type of digital intervention (web-based portals, social media, live chat with human staff or chatbots, mobile applications, video-assisted STBBI testing services, vending machines, virtual reality and/or a combination of the above)	
Type of sample collection model (Self-sample collection and interpretation, self-sample collection and postal, lab-assisted sample collection, Others indicate)	
Short description of the intervention (including target population of the intervention)	

Data Collection

What types of data are collected/managed/shared?	
How is this data collected/managed/shared? (describe the tool, if there is one)	

Data collection and sharing context (who is collecting data and who is it being shared with, how?)	

Study Design

Description of study design	
Theoretical framework for development and/or evaluation (describe, if there is one)	
Eligibility (inclusion/exclusion criteria)	
Control/comparison group	
Analyses conducted	
Outcome measures	
The study conducted sub-analysis or stratified effects by any of the PROGRESS-Plus criteria (yes/no)	

Study Findings (add more rows to elaborate on findings as needed)

Key descriptive statistics	
Primary outcomes (uptake of STBBI testing – e.g., completion and return of STBBI test samples, frequency of testing and repeat testing rates)	
Secondary outcomes: STBBI positivity rates, linkage to treatment and partner notification rates.	
Other self-reported outcomes	
Describe stratified outcomes or sub-analysis (if available)	

Assessment & Impacts

Strengths and Limitations (as noted by authors)	
MMAT Assessment Results	
Key impacts and lessons learned	

Key Quotes (for thematic analysis (if needed), add more rows as needed)

Notes	Quote
Design factors described as relevant to observed results	
Implementation factors descrived as relevant to observed results	

Iyamu I, *et al. Sex Transm Infect* 2023; 99:554–560. doi: 10.1136/sextrans-2023-055749

Appendix D: Consideration of the PROGRESS-Plus factors in included articles

**Author (year)**	**Name of digital intervention**	**Total number of participants**	**Place of residence**	**Race/Ethnicity**	**Occupation**	**Gender**	**Religion**	**Educational attainment/ level**	**Sexual orientation**	**Socio-economic status (or index of multiple deprivation)**	**Social capital**	**Age**	**Others (indicate)**
Anand 2017	Adam's Love (www.adamslove.org)	186	Bangkok<br>Others	NS	NS	NS	NS	Less than bachelor's degree<br>Bachelor's degree or higher	Gay<br>Bisexual<br>Male<br>Transgender<br><br>*Category is conceptually wrong.	Monthly income:<br></= 500 USD<br>501-1000 USD<br>1001-1500 USD<br>=/> 1500 USD	NS	Age in years	
MacGowan 2020	AIDSVU.org - (evaluated using the eSTAMP project)	2665	US Census region of residence:<br>Northeast<br>Midwest<br>South<br>West	Non-hispanic white<br>non-hispanic black<br>Hispanic<br>Others/mixed	Employment status<br>Employed<br>Unemployed	NS - Targeted intervention at only MSMs	NS	</= High school/GED<br>> High School	Homosexual/gay<br>Bisexual<br>Heterosexual/other<br>*Category is conceptually wrong.	NS	NS	18-29 years<br>>/= 30 years	Health insurance<br>Yes<br>No

Ricca 2016	Checking in	896	NS	White, non-Hispanic<br>Black, non-Hispanic<br>Hispanic	NS	NS	NS	College/post graduate<br>Some college/associate degree<br>High school or GED<br>Less than high school or unknown	Homosexual<br>Bisexual<br>Heterosexual<br>Other	Yearly income:<br><US $ 14999<br>US$15000-US$39999<br>US$40000-US$74999<br>>US$75000	NS	18-24 years<br>25-34 years<br>35-44 years<br>>/= 45 years	NS
vanBergen 2010	Chlamydia Screening Implementation (CSI)	261025	Amsterdam<br>Rotterdam<br>South Limburg	NS	NS	Men<br>Women	NS	NS	NS	NS	NS	16-19 years<br>20-24 years<br>25-29 years	NS

OpdeCoul 2012	Chlamydia Screening Implementation (CSI)	31466	Amsterdam Rotterdam	Ethnicity The Netherlands Surinman/ Antillean/ Aruban Turkish/Moroccan/North African Sub-Saharan African Western, other Non-Western, other	NS	Male Female	NS	Low Medium High	NS	High SES High to average SES Low to average SES Low SES	NS	16-19 years 20-24 years 25-29 years	NS
Gotz 2013	Chlamydia Screening Implementation (CSI)	4191	Rotterdam Amsterdam South Limburg	Ethnic Origin: Dutch Surinamese/Antillean Turkish/Moroccan Sub-Saharan Africa Other	NA	Male Female)	NA	Low Mid High Missing	NA	SES High High average Low average Low Missing	Community risk Low Medium High	16 to 19 years 20 to 24 years 25 to 29 years	NS

VanDen Broek 2012	Chlamydia Screening Intervention (CSI)	317304	Region:<br>Amsterdam<br>Rotterdam<br>South Limburg	Ethnic Origin:<br>Dutch<br>Surinamese/Antillean<br>Turkish/Moroccan<br>sub-Saharan African<br>Other	NS	Male<br>Female	NS	NS	NS	Considered in analysis but no table 1 figures	NS	16-19 years<br>20-24 years<br>25-29 years	Community risk<br>High<br>Medium<br>Low
Lydie 2017	Chlamyweb	7215	Size of the city<br><2000 inhabitants<br>From 2000 to 20,000 inhabitants<br>From 20,000 to 100,000 inhabitants<br>>100,000 inhabitants<br>Paris area	NS	Student<br>High School<br>Professional training<br>Employed<br>Unemployed<br>Others	Women<br>Men	NS	NS	Ever had a same sex partner<br>Yes<br>No	NS	NS	18-19 years<br>20-21 years<br>22-24 years	Place of birth:<br>Mainland France<br>France oversea<br>Maghreb<br>sub-Saharan Africa<br>Others

Iyamu I, *et al. Sex Transm Infect* 2023; 99:554–560. doi: 10.1136/sextrans-2023-055749

Kersaudy-Rahib 2017	Chlamyweb	11075	Size of the city:<br>Less than 2000 inhabitants<br>From 2000 to 20000 inhabitants<br>From 20000 to 100000 inhabitants<br>More than 100000 inhabitants<br>Paris area<br>Unknown	NS	Student<br>General high school<br>Professional high school<br>Professional training<br>Employed<br>Unemployed<br>Others	Biological sex<br>Men<br>Women	NS	NS	NS	NS	NS	Age in years	Place of birth:<br>France mainland<br>France overseas<br>sub-Saharan Africa<br>Maghreb<br>Others

Spielberg 2014	e-STI	217	County: Alameda San Francisco Contra Costa San Mateo	Non-Hispanic White Non-Hispanic African American Latina/Hispanic Other	NS	NA	NS	</= High School College or advanced degree	NS	Income $ <10831 10832 >43320 No answer	NS	18-21 years 22-25 years 26-30 years	Relationship status Married In a relationship No steady relationship
Fistonich 2021	GetChecked DC.org	1245	NS	Race Black Non-hispanic white Hispanic white Asian, native Hawaiian, other Pacific Islander American Indian/Alaska Native	NS	Cis-male Cis-female Trans-male Trans-female Other	NS	NS	MSM MSMW MSW WSM WSMW	NS	NS	Age in years	Insurance: Employer insurance Uninsured Medicare Medicaid Family member DC health link

Gilbert 2019	GetChecked Online.com	19497	NS	Intervention<br>White<br>Non-white/racialised<br>Missing	NS	Women<br>Men (extrapolated from the table)<br>Missing	NS	NS	Women*<br>MSM<br>MSW<br>Missing<br><br>*Women were not classified according to sexual orientation.	NS	NS	< 30 years<br>30-39 years<br>40+ years<br>Missing	NS

Zuure 2011	heptest.nl	1480	Region of residence: Amsterdam South Limburg Unknown<br><br>Proximity of the nearest laboratory (km) <1.17 - 319 (21.6%) 1.17 to 2.19 - 384 (25.9%) 2.20 to 6.08 - 362 (24.5%) >6.08 - 362 (24.5%) Unknown - 51 (3.5%)	NS	NS	Male Female	NS	Low Low-medium Medium-high High No answer	NS	NS	NS	<32 years 32-45 years 46-54 years >54 years Missing	Country of birth: Netherlands Elsewhere<br><br>Health insurance: Insured No/Unknown

Chan 2021	HIVST Online	350	NS	NS	NS	NS	NS	Secondary or below<br>College or above	Gay<br>Bisexual<br>Heterosexual<br><br>*Gay is gender and not sexual orientation	NS	NS	18-30 years<br>31-40 years<br>>40 years	Marital/cohabitation status:<br>Currently single<br>Cohabitate/ married with a man<br>Cohabited/ married with a woman
Jenkins 2011	iwantthekit.org (IWTK)	35980	NS	White<br>Black<br>Other or unknown<br>Missing	NS	Female<br>Male	NS	NS	NS	NS	NS	NS	NS
Gaydos 2013	Iwantthekit.org (IWTK)	912	West Virginia<br>Maryland<br>Baltimore<br>District of Columbia<br>Illinois<br>Colorado<br>Other states<br>Missing	Race:<br>White<br>Black<br>Other<br><br>Ethnicity:<br>Hispanic<br>non-hispanic<br>Missing	NS	NS	NS	Less than high school<br>High school<br>Community college<br>Some college<br>Bachelor's<br>Masters<br>Doctoral<br>Other<br>Missing	Partner's gender: Males only)<br>Females only<br>Males and Females<br>Missing	Income:<br><$10,000<br>$10,000-$49,999<br>$50,000-99,999<br>>$100,000<br>Missing	NA	15-19 years<br>20-24 years<br>25-29 years<br>>/= 30 years	Health insurance:<br>With<br>Without<br>Missing<br><br>Marital status:<br>Single<br>Married<br>Separated<br>Divorced<br>Missing

Yan 2020	Mailing rapid test reagent kit app	1315	NS	NS	NS	NA	NS	Junior high school or below<br>High school<br>University/ college or above	NS	NS	NS	</=25 years<br>26-40 years<br>>/=41 years	Marital Status:<br>Married<br>Unmarried<br>Divorced or widowed
Rahib 2021	MemoDépistages Programme	7158	Area of residence<br>Fewer than 2000 inhabitants<br>Between 2000 and 19999<br>Between 20,000 and 99,999 inhabitants<br>More than 100,000 inhabitants	NS	NS	NA	NS	High School or less<br>College or more	MSM	Perceived financial situation:<br>Good<br>Average<br>Bad	NS	25-42 years<br>under 30 years<br>30 years and over	Region<br>Paris area<br>Auvergne Rhone Alpes<br>Provence Alpes Cote d'Azur<br>Occitanie<br><br>Born in France<br>Yes<br>No
Phanuphak 2018	Not stated	571	NS	NS	Unemployed/Student<br>Employed<br>Service worker	Cisgender Men (MSM)<br>Transgender Women (TGW)	NS	Less than bachelor<br>Bachelor or above	Sexual orientation of TGW is not reported.	Income (USD)<br><429<br>429 to 857<br>858 to 1429<br>1430 to 2857	NS	18 to 25 years<br>> 25 years	NS

										=>2858			
Day 2021	Sexual Health London (SHL)	119329	NS	African<br>Asian<br>Caribbean<br>Other<br>White	NS	Cisgender female<br>Cisgender male<br>Transgender man<br>Transgender woman<br>non-binary/ gender fluid<br>Other	NS	NS	NS	NS	NS	NS	Sex Assigned at birth:<br>Male<br>Female<br>Intersex
Wilson 2017	SH:24	2063	NS	White<br>Black/African/Caribbean/ black British<br>Asian/Asian British<br>Mixed/multiple ethnicity<br>Other	NS	Female<br>Male<br>Transgender	NS	NS	MSM<br>Other<br>Refused	NS	NS	16-19 years<br>20-24 years<br>25-30 years	

Barnard 2018	SH:24	5747	NS	White British<br>White other<br>Black African<br>Black Caribbean<br>Black Other<br>Mixed white, black African or Caribbean<br>South Asian<br>Any other ethnic group<br>Not stated	NS	Male<br>Female	NS	NS	Heterosexual<br>Homosexual<br>Bisexual<br>Unknown	IMD quintile<br>1 (most deprived)<br>2<br>3<br>4<br>5 (least deprived)	NS	16-20 years<br>20-25 years<br>25-30 years<br>30-35 years<br>35+ years	Tested positive for chlamydia and gonorrhoea
Wilson 2019	SH:24 (www.sh24.org.uk)	528	NS	White/White British<br>Black/Black British<br>Asian/Asian British<br>Mixed/Multiple Ethnicity<br>Other	NS	Cisgender Women<br>Cisgender Men<br>Transgender Men & Women	NS	NS	MSM<br>Other<br>Refused	NS - Index of Multiple Deprivation used in analysis but not included in table 1.	NS	Age in years	NS

Grov 2019	Together 5000	8777	NS	White<br>Black or African American<br>Latinx<br>Asian or Pacific Islander<br>Multiracial/Other	Working full-time<br>Working part-time<br>Working or full-time student<br>Unemployed/other	Cisgender man<br>Trans Woman<br>Trans Man<br>non-binary (male at birth)	NS	Less than high school diploma<br>High school diploma or GED<br>Some college or associate degree<br>College graduate or higher	Gay/Queer/Homosexual<br>Bisexual<br>Other non-heterosexual	Income<br><$20,000 USD<br>$20,000 - $49.999 USD<br>>$50,000 USD	NS	Age in years	Housing instability<br>Yes within the last 5y<br>No or not within the last 5y<br><br>Transactional sex (last 3 months)<br>Yes<br>No
Banerjee 2018	Umbrella	No of patients using online testing - 9258, No of patients using clinic - based testing - 19193	NS	Ethnicity<br>White<br>Black/British Black<br>Asian/British Asian<br>Other<br>Not specified	NS	Female<br>Male<br>Transgender	NS	NS	Heterosexual male<br>Heterosexual female<br>MSM<br>Transgender<br>Bisexual<br>WSW	NS	NS	16-24 years<br>>/= 25 years	NS

Manavi 2017	Umbrella health	5310 kits	NS	NS	NS	Female<br>Male<br>Transgender (Female to male)<br>Transgender (Male to Female)	NS	NS	NS	2015 IMD rank<br><5000 - 1855<br>5000-14999<br>15000+	NS	NS	Country of Birth<br>Respondent born outside Northern EU<br>Yes<br>No
Wu 2021	Xutong's public WeChat account	652	Rural<br>Urban	Han<br>Other minorities	Employment status<br>Employed<br>Unemployed	Gender Identity<br>Male<br>Female<br>Transgender<br>Unsure/other	NS	High school or less<br>Some college<br>University	Gay<br>Heterosexual<br>Bisexual<br>Unsure	Annual income (US dollars)<br>Index Men<br><2518<br>2518 - 5038<br>5039 - 8393<br>8394 - 13430<br>>13430	NS	Age in years	Marital status:<br>Never married<br>Engaged or married<br>Separated, divorced, or widowed

Appendix E: Characteristics of included articles and outcomes reported

**Author (year)**	**Name of digital intervention**	**Type of digital intervention**	**Type of sample collection model**	**Study aims/research question(s)$**	**Key results (primary outcomes)**	**Key results (secondary outcomes)**
Anand 2017[29]	Adam's Love (www.adamslove.org)	Video-assisted testing; Web-based testing; Electronic health records	Self-sample collection and interpretation	To examine factors associated with uptake and utilization of Adam's love among MSM/Transgender people (comparing web testing vs hybrid vs clinic-based testing)	Participants using web-based testing were more likely to be younger (median age - 25 years), bisexual, had never tested for HIV or refused to answer, or tested more than 1 year ago. Participants with income of ≤ 500 USD or 501-1000 USD had increased odds for using web-based testing records at least twice vs participants earning ≥1500 USD per month.	HIV prevalence was highest among participants in the web-based testing arm compared with hybrid and clinic-based testing.
MacGowan 2020[31]	AIDSVU.org - (evaluated using the eSTAMP project)	Web-based testing	Self-sample collection and interpretation	To evaluate the effect of providing HIV-self tests on frequency of testing, diagnoses of HIV infection and sexual risk behaviours	Program users reported testing more frequently than control participants (5.3 vs 1.5 tests per 12 months; *P*< 0.001) and 76.6% of program users (777/1014) reported testing ≥ 3 times in 12-months compared with 22% (215/977; P< 0.001) of controls.	HIV positivity was higher among users than controls at 3 (0.9% vs 0.1%) & 12-months (1.9% vs 0.8%).
Ricca 2016[52]	Checking in	Web-based testing	Self-sample collection and postal	To describe factors associated with returning home-based specimen collection kits for HIV testing among participating MSM	Among 896 participants, 82% returned test kits. The adjusted odds of black non-Hispanic men returning a test kit were half of the odds of white, non-Hispanic men (aOR: 0.49; 95%CI: 0.31-0.78). The odds of returning a test kit were higher among participants with some college education (aOR: 1.67;95%CI: 1.03-2.69) or a college degree (aOR: 1.76; 95%CI:1.01-3.06) compared with high school or general educational development educated participants.	HIV positivity rate was 3.4%. Of 25 men with HIV-positive results, 11 were white, non-Hispanic (2% seropositivity), 9 were black, non-Hispanic (8% seropositivity), and 5 were Hispanic (4% seropositivity).
van Bergen 2010[43]	Chlamydia Screening Implementation (CSI)	Web-based testing	Self-sample collection and postal	To describe the register-based, systematic, selective screening programme in which the internet plays a major role.	11.9% of invitees requested a kit within 4 weeks of invitation and 8.3% additionally requested after reminders. Participation rates were higher among women (21.0%) compared with men (10.4%), among those 20 years old and older (17.6%) than among those less than 20 years old (9.9%), in urban areas (Amsterdam and Rotterdam at 17.0% and 15.2% respectively) than the semi-rural region (11.0%).	Chlamydia positivity rate was 4.2% (95% CI 4.0-4.4%). Positivity rate was lower among men than women, but higher among people <20 years (7.3%) compared with those ≥ 20 years (3.8%).

Op de Coul 2012[44]	Chlamydia Screening Implementation (CSI)	Web-based testing	Self-sample collection and postal	To examine whether the CSI reached the right populations for screening.	Adjusted participation rates were higher in females (25.1%) than males (12.9%) but were lowest among 16–18-year-old people and highest among women aged 26 (28.0%) and 27 years (28.4%) respectively. Women were twice as likely to participate as men in all subgroups of age (range: female [F], 14%–24%; male [M], 6%–13%) and ethnicity (F, 18%–29%; M, 7%–14%), except for Turkish/Moroccan women (F, 5.7%; M, 5.4%). Dutch invitees of both sexes had higher participation rates than non-Dutch. People in medium-/high community risk areas, and areas with low SES had significantly lower participation.	Positivity rates were highest in Surinamese / Antillean men (9.7%) and women (8.4%) and sub-Saharan African women (9.2%). Ethnic groups with highest positivity participated less (14%–16%) than Dutch people (21%). Positivity rates in 16–19-year-old girls was 8.0%
Gotz 2013[45]	Chlamydia Screening Implementation (CSI)	Web-based testing	Self-sample collection and postal	To describe the prevalence of repeat infection during three consecutive annual screening rounds and study participation in retesting.	Retest participation was lower in men compared with women (OR 0.85, 95%CI 0.71-1.02), lowest among 16-19- year-olds (OR 0.7, 95%CI 0.56-0.87), and lower in non-Dutch people - Surinamese/Antillean (OR 0.89, 95%CI 0.62-0.92) and Sub-Saharan African (OR 0.62, 95%CI 0.45-0.68). Low and middle education level was significantly associated with low participation (low OR 0.61, 95% CI 0.45-0.82, middle OR 0.83, 95% CI 0.7-1.0).	Retest positivity was higher in in the young, highest (14.6%) in 16–19-year-olds, higher among participants with lower education (15.2%), higher in Surinamese /Antillean (13.1%), Turkish /Moroccan (12.9%), and Sub-Saharan African people (18.6%) compared with the Dutch (6.6%).
van Den Broek 2012[46]	Chlamydia Screening Intervention (CSI)	Web-based testing	Self-sample collection and postal	To investigate the effects of yearly invitations for Chlamydia screening on the percentage of positive Chlamydia test results (positivity) and uptake of chlamydia screening.	Women were more likely to participate than men in all three rounds (OR 2.34, 95%CI 2.31-2.38)). Participation rates were higher among older age groups (OR 1.89, 95%CI 1.85-1.92) for 25–29-year-old vs 16–19-year-old), Dutch invitees (OR 1.90, 95%CI 1.87-1.92) vs non-Dutch, in people living in clusters of low community risk (OR 1.57, 95%CI 1.54-1.61) vs high risk areas, and for people living in areas with high socioeconomic status scores (OR 1.28, 95%CI 1.24-1.33) vs low socioeconomic status. Similar trends occurred in all screening rounds.	Chlamydia positivity rate was 4.3% (1851/43358), 4.0% (1153/28803), and 4.1% (981/23899) in successive rounds. Positivity rates were higher among women than men, among young people under 20 years, people in areas with low socioeconomic status, and in non-Dutch people.

Lydie 2017[47]	Chlamyweb	Web-based testing	Self-sample collection and postal	To examine factors that influence acceptance of self-screening kits supplied to participants	Among 7215 participants, 61.8% returned test kits with more women returning than men (63.5% vs 59.2%). Participation rate was higher in women (33.1%) than in men (23.9%). Among women, compared to those born in Mainland France, users born in Maghreb were less likely to return kits (aOR 0.09, 95% CI 0.01-0.76). Among men, compared with students, employed (aOR 0.65, 95%CI 0.49-0.87), and unemployed (aOR 0.69, 95%CI 0.49-0.98) people were less likely to return kits.	NS
Kersaudy-Rahib 2017[37]	Chlamyweb	Web-based testing	Self-sample collection and postal	To evaluate whether the offer of home-based testing via the internet increased the number of young people tested for chlamydia compared with the current testing strategy	Testing rates were higher in the intervention compared with primary care (RR 3.37, 95%CI 3.05-3.74). The rates were significantly lower among men than among women (23.9% vs 33.9%) in the intervention group and 5.3% vs 11.6% in the controls. The intervention effect was higher in men (RR 4.55, 95%CI 3.77-5.49), than in women (RR 2.94, 95%CI 2.60-3.33). Among women, testing rates were positively correlated with age. In the intervention, testing rates differed by place of birth: 47.6% of French Caribbean women screened compared with 33.6% of women born elsewhere.	Positivity rate was 6.8% (110/1616) in the intervention group and 6.3% (30/480) in the control group with no significant difference. Positivity rate was higher in females than in males (8.3% vs 4.4%; p<0.001) in the intervention arm.
Spielberg 2014[53]	e-STI	Web-based testing	Self-sample collection and postal	To examine the acceptability, feasibility of a fully integrated online system (eSTI) for STI testing, treatment, and linkage to care	Of 217 participants, 143 (67%) returned kits to the lab. Non-Hispanic African Americans, compared with non-Hispanic whites (aOR 0.29, 95%CI 0.09-0.82) and participants with high school education or less, vs. those with college/advanced degrees (aOR 0.22, 95%CI 0.10-0.44) were less likely to return kits.	Positivity rate was 5.6% (95% CI 2.5-10.7%) for new STIs.
Fistonich 2021[36]	GetCheckedDC.org	Web-based testing	Self-sample collection and interpretation; Self-sample collection and postal	To evaluate the usage and public health impact of free, delivered rapid HIV self-tests and mail-in STI self-testing in an economically and racially diverse city and to compare demographic characteristics of participants using the service.	Overall, 1,245 requests were made with delivery of 1,089 rapid HIV and 1,262 mail-in STI self-tests. Compared with non-Hispanic whites, blacks were less likely to return test kits (RR 0.60, p<0.001). Compared with those with employer insurance, those uninsured (RR 0.62, p<0.001), on Medicare (RR 0.57, p=0.04), on Medicaid (RR 0.58, p<0.001) and supported by family (RR: 0.74, p=0.027) were less likely to return test kits. MSM were more likely to return test kits (RR 1.29, p=0.037). Those who returned tests were less likely to have a residence within both the third and fifth highest morbidity ZIP codes for gonorrhoea and chlamydia (RR 0.72, p=0.009 and RR 0.80, p=0.007).	Positivity rate - 19 (7.2%), CT - 13 (4.94%) and GC - 7 (2.66%)

Gilbert 2019[38]	GetCheckedOnline.com	Web-based testing	Lab-assisted sample collection	To determine whether the use of GCO is associated with higher rates of repeat testing among individuals in Vancouver, Canada.	Compared to STI clinic clients, GCO clients included more individuals 30-39 years of age, white people, women, MSW, clients with prior STI and clients reporting HIV-positive partners (all p<0.05). The unadjusted relative rate (aRR) of repeat testing was 1.22 (95%CI: 1.14-1.31) among GCO clients vs. clinic clients. The aRR of repeat testing for GCO clients was significantly higher on at 1.26 (95% CI: 1.15-1.37).	
Zuure 2011[50]	heptest.nl	Web-based testing	Lab-assisted sample collection	To describe the HepTest project and evaluate its usage, determinants of usage, and clinical outcomes.	Of 1480 eligible for the blood test, 420 opted for testing (28%). Individuals of older age, higher educational level, and with residence in a less urbanized region were more likely to be tested. Not having health insurance was associated testing.	HCV positivity rate was 3.6%.
Chan 2021[30]	HIVST Online	Web-based testing	Self-sample collection and interpretation	To evaluate the effectiveness of HIVST-online in increasing testing and repeated testing among MSM in Hong Kong	Among 350 users, 48.3% of users received HIVST. Ever using HIVST online at baseline was associated with higher uptake of HIVST- online (OR: 2.53, 95% CI: 1.61-3.98). No sociodemographic factors predicted uptake of HIVST-online.	Four HIVST-online users were screened to be HIV positive. All of them received the appropriate treatment services.
Jenkins 2011[42]	iwantthekit.org (IWTK)	Web-based testing	Self-sample collection and postal	To determine if IWTK would identify infected individuals who might not otherwise be detected by traditional means.	Overall, 343 kits were requested, of which 137 (39.9%) were returned and tested. Internet-based testing attracted more males (23.4% vs 37.7%). While 32.1% of testers in traditional setting were black, 22.5% were black on the internet-based testing but the number of missing was higher on the internet-based testing compared with traditional setting (6.5% vs. 0.7%). Racial distribution among males was significantly different, with a greater proportion of white males utilizing the internet compared to traditional means (69.2% vs. 46.4%), while the ratio of black males was lower (19.2% vs 49.9%).	Total CT positivity rates for internet and traditional means were not significantly different.
Gaydos 2013[51]	Iwantthekit.org (IWTK)	Web-based testing	Self-sample collection and postal	To ascertain re-screening practices in the home collection programme of IWTK for CT, GC, TV.	Repeat IWTK users were more likely to be aged ≥20 years (OR 2.10, 95%CI:1.30-3.38), reside in Maryland (OR 2.03, 95%CI:1.31-3.13), have been treated for an STI (OR 2.32, 95%CI: 1.57, 3.44), have been treated for trichomonas (OR 2.33, 95% CI: 1.46, 3.71).	Positivity of first-time testers was 15.8%, and for repeat-testers at second visit was 13.2%.
Yan 2020[35]	Mailing rapid test reagent kit app	Mobile applications	Self-sample collection and interpretation	To examine the adherence to HIV testing among MSM, as well as the impact factors and potential effects of their adherence.	Among 1315 participants, 131 (9.96%) were adherent to recommended testing frequency, while 1184 (90.04%) were non-adherent. Unmarried MSM were more likely be adherent (aOR 2.31, 95%CI 1.13-4.71), and 62.07% (1677/2702) of registered users of the app	At 1-year follow-up, 20 seroconversions were found, including 8 in the adherence and 12 in the nonadherence groups.

					received the HIVST service and uploaded test results.	
Rahib 2021[55]	MemoDépistages Programme	Web-based testing	Self-sample collection and postal	To assess different factors that impact the overall participation in the first screening round of the programme	Overall, 3428/7158 men (47.9%) accepted kits, among whom 1948 (56.8%) returned samples. Sample return was lower for men who had not attended college (aOR 0.65, 95%CI 0.56-0.76), those reporting difficult financial situation (aOR 0.7, 95%CI 0.58-0.86 compared with those with a good situation. Participation rates were lower for men ≥ 30 years (aOR=0.66, 95%CI 0.58- 0.75) vs those <30. No differences were found by place of residence.	NS
Phanuphak 2018[54]	Not stated	Video-assisted testing services	Self-sample collection and interpretation	To explore characteristics of Thai MSM and Transgender women (TGW) and factors they considered when choosing online, offline, and mixed HIV counselling services.	Being TGW (OR 6.66, 95% CI 2.91-15.25) and having preference towards online services (OR 5.73, 95% CI 2.99-10.98) and home-based HIV testing (OR 6.00, 95% CI 3.1-11.63) increased participants' likelihood to choose online HIV testing and post-test counselling. No difference was found in other PROGRESS criteria in the multivariate analysis.	
Day 2021[41]	Sexual Health London (SHL)	Web-based testing	Self-sample collection and postal	To assess the sexual health needs, practices, STI/HIV positivity, and satisfaction rates of trans and non-binary people in a large STI screening e-service.	There was no difference in kit return rates between Trans and non-binary (TNB) and cisgender individuals (OR 1.00 95% CI 0.81-1.24). TNB users were more likely to return blood samples than cisgender users (OR 1.6 95%CI 1.06-2.36). Similar proportion of TNB and cisgender users received testing after registration.	STI positivity rates were higher among TNB vs. cisgender users for all STBBIs.
Wilson 2017[39]	SH:24	Web-based testing	Self-sample collection and postal	To assess the effectiveness of an e-STI testing and results service (chlamydia, gonorrhoea, HIV, and syphilis) on STI testing uptake and STI cases diagnosed.	At 6 weeks, 50.0% of the intervention group had completed an STI test compared to 26.6% in the control group (RR 1.87, 95%CI 1.63-2.15); 2.8% of the intervention group vs 1.4% in the control group had been diagnosed with an STI (RR 2.10, 95%CI 0.94-4.70). The proportion of participants treated was 1.1% in the intervention group vs 0.7% in the control group (RR 1.72, 95%CI 0.71-4.16). Time to test was shorter in the intervention group compared with the control group (28.8 days vs 36.5 days). No differences were observed for time to treatment (83.2 days vs. 83.5 days). STI testing was increased in all sub-groups with no outcomes difference between sub-groups.	Among testers, 4.3% (19/439; 95%CI 2.8 to 6.7) tested positive for an STI in the intervention group and 4.6% (8/173; 95%CI 2.3 to 9.0) tested positive for an STI in the control group. Median time from diagnosis to treatment was 2 days in the intervention group and 4 days in controls.

Wilson 2019[40]	SH:24 (www.sh24.org.uk)	Web-based testing	Self-sample collection and postal	To examine the effect of e-STI testing on uptake of any STI test, uptake of chlamydia and gonorrhea tests and time to test, among a subsample of trial participants who reported never having tested for an STI at baseline	Overall, 45.3% of people in the program completed at least one STI test, vs. 24.1% of the controls (RR 1.88, 95% CI 1.47- 2.40). 44.3% of people in the program tested for chlamydia and gonorrhea combined, vs. 24.1% in the control (RR 1.84, 95% CI 1.44-2.36). The intervention reduced time to test for any STI at 42 days, but not at 7 and 14 days. The intervention was more effective in those recruited via face-to-face settings vs. those recruited online or via other methods (e.g., print media). No heterogeneity was observed across any other subgroups.	Among those who completed a test, 4.3% (4/94) of the intervention arm tested positive for an STI, compared with 2.3% (1/44) in the control.
Grov 2019[32]	Together 5000	Web-based testing	Self-sample collection and postal	To describe factors associated with returning HIV self-collection kits in a large, ongoing Internet-based U.S. national cohort of men, transmen, and transwomen who have sex with men.	For every 5 years increase, odds of returning kits increased aOR1.16 (95%CI 1.1-1.22). Vs. white men, Black and African American participants were less likely to return an HIV test kit (aOR 0.71, 95%CI 0.58-0.88). Vs. Cis men, transwomen were less likely to return HIV test kits (aOR 0.33, 95%CI 0.17-0.65). College graduates had higher increased odds of returning kits (aOR 1.61, 95%CI 1.29-2.00).	NS
Banerjee 2018[33]	Umbrella	Web-based testing	Self-sample collection and postal	To compare home-based to clinic-based testing in terms of patient demographics (age, gender, sexuality, and ethnic origin) in the UK	Home-based online testing was more popular than clinic-based testing for patients aged 16-24 years (65% vs 50%), white (72% vs 42%), heterosexual female (65% vs. 56%) and asymptomatic (80% vs. 51%). Home-based testing was less popular in ethnic minorities like Black/British Black (10% vs. 21%) and Asian/British Asian (6% vs. 11%) patients. Overall, 48% of people requesting tests online returned the kits. Vs. clinic-based patients, those returning home-based test kits were more likely to be aged 16-24 years (64% vs.50%), heterosexual female (69% vs. 56%), White (75% vs. 42%) and asymptomatic (82% vs. 51%)	Positivity rate was higher in clinic- vs. home-based testing group (10%vs. 8%). Treatment rate was lower in the home- vs. clinic-based testing group (46% vs. 88%). Median time to treatment was lower in the clinic vs. home-based testing group for CT (3 vs. 6 days) and GC (0 vs. 6 days)
Wu 2021[34]	Xutong's public WeChat account	Social media	Self-sample collection and interpretation	To assess a social media based secondary distribution of HIV/Syphilis self-testing among Chinese MSM	No significant differences in sociodemographic characteristics (age, marital status, residence, education, employment status and annual income) between those who return tests and those who did not.	Overall, 20 of 612 unique testers (3%) had a reactive HIV self-test result.

Manavi 2017[49]	Umbrella health	Web-based testing	Self-sample collection and postal	To investigate the return of home sampling kits after registration with an online health website in the cities of Birmingham and Solihull	About 58.4% (3099) of requested kits were returned to the lab. Women were more likely than men to return kits (61.2% vs 53.1%). Among males, users requesting MSM kits had similar rates of kit return to females, but heterosexual men were significantly less likely to return kits. Users returning kit were in less deprived areas. Users born within and with partners within UK/northern EU had higher return rates vs. those born outside northern EU. Patients reporting symptoms were also less likely to return kits, (aOR 0.77, 95%CI 0.67 to 0.89, vs asymptomatic patients). Per 10000 ranks in IMD score increase, odds of kits return increased (aOR 1.08, 95% CI 1.01-1.15).	NS
Barnard 2018[48]	SH:24	Web-based testing	Self-sample collection and postal	To compare the characteristics of people completing STI testing using an online service for self-sampling to those using clinic services in the two neighbouring boroughs.	Users more likely to return kits included those 20-25 and 25-30 years compared with 16-20-year-olds, women, white British people, homosexual and bisexual vs heterosexual users, residents of areas with less deprived IMD quintiles. Among women, being mixed white, black African or Caribbean was not associated with lower odds of using online services but was for men. Among women, being homosexual increased the likelihood of online service use more than it did for men. Compared with white British individuals, black African, mixed white, black African or Caribbean, any other ethnic group were less likely to return a sample for testing. Compared with 16–20-year-olds, 20-25 years, 25-30 years, 30-35 years, and 35+ were more likely to return kits.	NS

[$] Study aims have been paraphrased to be more concise; MSM - Men who have sex with men; CI – Confidence Interval; GC – Gonorrhea; CT – Chalmydia Trachomatis; TV – Trichomonas Vaginalis; NS – Not specified; Note – references linked to main document bibliography.
